# Accurate Prediction of Antimicrobial Susceptibility for Point‐of‐Care Testing of Urine in Less than 90 Minutes via iPRISM Cassettes

**DOI:** 10.1002/advs.202303285

**Published:** 2023-08-16

**Authors:** Xin Jiang, Talya Borkum, Sagi Shprits, Joseph Boen, Sofia Arshavsky‐Graham, Baruch Rofman, Merav Strauss, Raul Colodner, Jeremias Sulam, Sarel Halachmi, Heidi Leonard, Ester Segal

**Affiliations:** ^1^ Department of Biotechnology and Food Engineering Technion – Israel Institute of Technology Haifa 3200003 Israel; ^2^ Department of Urology Bnai Zion Medical Center Haifa 3104800 Israel; ^3^ Department of Biomedical Engineering Johns Hopkins University Clark 320B, 3400 N Charles St Baltimore MD 21218 USA; ^4^ Department of Mechanical Engineering Technion – Israel Institute of Technology Haifa 3200003 Israel; ^5^ Laboratory of Clinical Microbiology Emek Medical Center Afula 1834111 Israel; ^6^ The Rappaport Faculty of Medicine Technion – Israel Institute of Technology Haifa 3200003 Israel; ^7^ Present address: Potomac Photonics, Inc 1450 S. Rolling Rd Halethorpe MD 21227 USA

**Keywords:** antibiotic resistance, antimicrobial susceptibility testing, bacteria, diffraction gratings, machine learning, optical sensors, urinary tract infecion

## Abstract

The extensive and improper use of antibiotics has led to a dramatic increase in the frequency of antibiotic resistance among human pathogens, complicating infectious disease treatments. In this work, a method for rapid antimicrobial susceptibility testing (AST) is presented using microstructured silicon diffraction gratings integrated into prototype devices, which enhance bacteria‐surface interactions and promote bacterial colonization. The silicon microstructures act also as optical sensors for monitoring bacterial growth upon exposure to antibiotics in a real‐time and label‐free manner via intensity‐based phase‐shift reflectometric interference spectroscopic measurements (iPRISM). Rapid AST using clinical isolates of *Escherichia coli* (*E. coli*) from urine is established and the assay is applied directly on unprocessed urine samples from urinary tract infection patients. When coupled with a machine learning algorithm trained on clinical samples, the iPRISM AST is able to predict the resistance or susceptibility of a new clinical sample with an Area Under the Receiver Operating Characteristic curve (AUC) of ∼ 0.85 in 1 h, and AUC > 0.9 in 90 min, when compared to state‐of‐the‐art automated AST methods used in the clinic while being an order of magnitude faster.

## Graphical abstract

A device for rapid phenotypic antimicrobial susceptibility testing by combining surface architecture favored by bacteria with optical spectroscopic techniques is presented. Resistance/susceptibility can be accurately determined for urinary tract infections within 90 min from clinical isolates or unprocessed urine with >90% accuracy and specificity. Employing a machine learning predictive algorithm further shortens the analysis time to just 1 h.

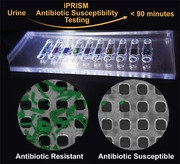

## 1 Introduction

According to the Centers for Disease Control and Prevention Antibiotic Resistance Threats Report (2019),^[^ ^1^ ^]^ >2.8 million antibiotic‐resistant infections with 35 000 deaths occur each year in the U.S. alone. These numbers are on the rise,^[^ ^2^, ^3^ ^]^ and the COVID‐19 pandemic^[^ ^4^, ^5^, ^6^ ^]^ may further intensify the emergence, transmission, and burden of AMR. The increasing frequency of resistance among human pathogens^[^ ^1^, ^7^, ^8^, ^9^ ^]^ is mainly attributed to the extensive and improper use of antibiotics, while the pace at which novel antibiotics are discovered has slowed drastically.^[^ ^10^, ^11^ ^]^ Thus, there is an immense need for rapid diagnostic methods for bacterial detection, identification, and more importantly, antimicrobial AST that would allow optimal pathogen‐targeted therapy and de‐escalation of unnecessary broad‐spectrum antibiotic use.^[^ ^3^, ^12^, ^13^, ^14^ ^]^

Current AST methods used in clinical microbiology laboratories include manual AST, such as broth or agar dilutions, disk diffusion, and gradient diffusion tests (Epsilometer test). For these methods, the minimum inhibitory concentration (MIC)^[^ ^15^, ^16^, ^17^, ^18^, ^19^, ^20^ ^]^ of the antibiotic agent is determined and is later translated into a susceptibility testing category based on criteria for different bacterial strains established by the Clinical & Laboratory Standards Institute (CLSI) in the USA and the European Committee on Antibiotic Susceptibility Testing (EUCAST) in Europe.^[^ ^21^, ^22^ ^]^ Although effective, these time‐consuming and laborious methods hinder targeted antibiotic therapy. Furthermore, it should be emphasized that these conventional AST methods require a pure culture, containing a single strain of a single species isolated from its original matrix,^[^ ^19^ ^]^ and as such necessitate a lengthy overnight cultivation and isolation prior to the initiation of susceptibility testing.^[^ ^23^ ^]^

To reduce manual sample handling, automated AST instruments have been developed and approved by the U.S Food and Drug Administration. These automated systems include the VITEK 2 system (bioMérieux, France, 2000), Sensititre ARIS 2X (Thermo Fisher Scientific, USA, 2004), MicroScan WalkAway systems (Beckman Coulter, a Danaher Corporation, USA, 1980), and BD Phoenix automated microbiology system (BD, USA, 2001). The use of automated systems increases the accuracy of the results and enables routine testing of a massive number of samples. However, the run time is still typically greater than 8 h and also requires pure bacterial isolates derived from an overnight culturing step.^[^ ^17^, ^19^, ^20^, ^23^, ^24^, ^25^ ^]^

Increasing efforts have been devoted to the development of innovative technologies for rapid AST. These include methods such as single‐cell optical imaging for tracking changes in motility and morphology, which can reduce the AST time to 30–120 min.^[^ ^26^, ^27^, ^28^, ^29^ ^]^ Yet, these methods rely on bacteria entrapment in microfluidic channels or hydrodynamic arrays and thus do not allow the formation of bacterial networks,^[^ ^19^, ^30^, ^31^, ^32^ ^]^ which may induce artifacts from a different bacteria stress response.^[^ ^33^, ^34^ ^]^ These methods also require the use of expensive equipment for high‐resolution imaging, which may restrict their routine application in a clinical laboratory setting. Other innovative methods are based on Raman spectroscopy,^[^ ^35^, ^36^, ^37^ ^]^ electrochemical impedance spectroscopy,^[^ ^38^, ^39^, ^40^, ^41^, ^42^, ^43^, ^44^ ^]^ respiration electroanalysis,^[^ ^45^ ^]^ field effect transistor,^[^ ^46^ ^]^ plasmon resonance,^[^ ^30^, ^47^ ^]^ fluorescence resonance,^[^ ^48^ ^]^ optically induced dielectrophoresis,^[^ ^49^ ^]^ nucleic acid amplification,^[^ ^50^, ^51^ ^]^ light‐scattering and diffractions techniques,^[^ ^52^, ^53^, ^54^, ^55^, ^56^, ^57^ ^]^ fluorescence or other metabolic activity indicators,^[^ ^58^, ^59^, ^60^, ^61^ ^]^ and entropy‐based image analysis.^[^ ^62^ ^]^ However, these methods often involve sample preparation steps, complicated readouts analysis, expensive equipment and labeling probes, analysis of unrepresentative sample populations or of secondary indicators of growth. Importantly, so far only a few AST methods can be performed directly on clinical samples.^[^ ^52^, ^63^, ^64^ ^]^

We have previously demonstrated that phase‐shift reflectometric interference spectroscopic measurements (PRISM) enable monitoring of bacteria colonization and antibiotic susceptibility within silicon diffraction gratings.^[^ ^65^ ^]^ These silicon micro‐structured arrays were found to provide both a preferable solid−liquid interface for bacteria settlement and an optical transducing element for monitoring bacterial growth upon exposure to antibiotics for real‐time and label‐free phenotypic AST.^[^ ^65^, ^66^ ^]^ In this work, we demonstrate intensity‐based PRISM (iPRISM) AST through an easy‐to‐use prototype device to different bacterial species and demonstrate its capability to perform AST directly on clinical urine samples from urinary tract infections (UTIs) within 90 min. Using *E. coli* and *Staphylococcus aureus* (*S. aureus*) as model bacteria, we apply a methodological study for the establishment of the iPRISM method for differentiation between susceptible (S) and resistant (R) strains in accordance with CLSI guidelines. Using standard American Type Culture Collection (ATCC) strains and a large dataset of clinical isolates from UTI and bloodstream infections (BSI), we determine the iPRISM threshold values in correlation to results obtained in the clinic. Furthermore, we demonstrate the capability of the method for fast differentiation between R/S strains within 90 min directly in urine samples without any pre‐processing steps. Notably, when using machine learning algorithms this time can be further reduced to only 1 h.

## 2 Results

### 2.1 PRISM Assay Design and Data Acquisition

**Figure 1:**
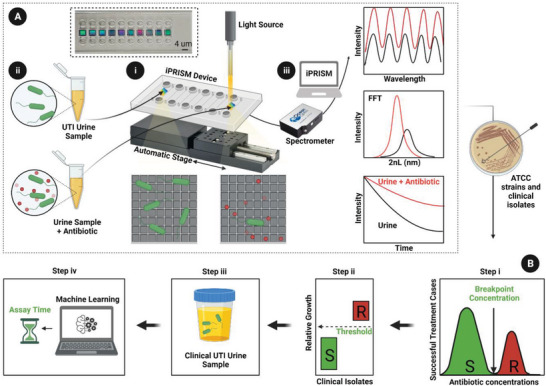
Concept of iPRISM AST assay. Schematic illustration of the iPRISM phenotypic AST assay concept and steps followed for direct testing of urine. A) The iPRISM method: i) Microstructured Si photonic grating chips are integrated into a custom‐made microfluidic device (cassette), which is fixed on an automated stage, allowing the screening of multiple samples. ii) Bacteria samples (in growth medium or in urine) with increasing antibiotic concentrations are introduced into the different channels. iii) The chips are illuminated with white light and the reflected light is collected with a CCD spectrometer. The resulting reflectance spectrum (upper graph) is analyzed with a Fast Fourier Transformation (FFT), resulting in a single peak (middle graph), in which intensity and position are sensitive to the bacteria colonizing the Si surface. Monitoring intensity changes over time indicates bacterial growth due to light scattering effects by the bacterial cells (bottom graph). Bacteria growth inhibition by an effective antibiotic concentration will show a smaller decrease in the intensity due to less light scattering by the fewer bacterial cells on the surface (red curves). B) The iPRISM method is used to establish a phenotypic AST assay, using standard ATCC strains and clinical isolates. Step i – antibiotic breakpoint concentrations produced by the CLSI to differentiate between S and R bacteria are verified in the iPRISM method with clinically relevant ATTC susceptible strains of *E. coli* and *S. aureus*. Step ii – The threshold values of the relative bacterial growth in the iPRISM method are established with clinical bacteria isolates to enable the differentiation between S and R strains. Step iii – The iPRISM phenotypic AST assay is demonstrated in clinical urine samples. Step iv– Using a machine learning algorithm the iPRISM assay time is optimized. Images adapted from BioRender.com.

While PRISM measurements from our previous works were acquired with an in‐house system,^[^ ^65^ ^]^ in order to safely handle clinical samples, we designed devices that can be commercially fabricated for single‐use testing of pathogenic bacteria and urine samples. These cassettes are fabricated from poly(methyl methacrylate) and are comprised of ten microfluidic channels, each integrated with a photonic silicon (Si) chip, as shown in **Figure** **1**A‐i. The fabrication of this cassette from an inexpensive and biocompatible thermoplastic ensures commercial viability and scalability, contrary to many devices constructed from polydimethylsiloxane.^[^ ^27^, ^61^, ^67^ ^]^

The iPRISM method is based on real‐time optical monitoring of bacteria colonization and growth on the Si diffraction gratings in the presence and absence of antibiotic agents (Figure 1A) and simultaneous analysis of their susceptibility/resistance profile. Each diffraction grating (termed as photonic chip) is used as an optical sensor, where changes in its reflectance are monitored over time. The individual Si chips are integrated into disposable plastic cassettes, each containing ten chips interfaced with individual channels (Figure 1A‐i). In a typical iPRISM assay, bacterial suspensions are pre‐mixed with different antibiotic concentrations and are introduced into separate iPRISM cassette channels (Figure 1A‐ii). We use bacterial suspensions according to the CLSI guidelines at final turbidity of 0.5 McFarland, which corresponds to an optical density value at 600 nm (OD_600_) of 0.1 in our studies. Pre‐mixing with antibiotics is carried out in a two‐fold dilution manner, similar to MIC determination by broth microdilution testing.^[^ ^68^ ^]^

Each individual photonic chip consists of periodic arrays of square microwells with a width of ∼2.7 µm, a depth of 4 µm, and a wall thickness of 0.7 µm (see Figure S1, Supporting Information). The photonic chips are amine‐functionalized to exhibit a slightly positive surface charge with a zeta potential of 53 mV.^[^ ^69^ ^]^ These conditions have been found to promote the bacteria capture on the chip's surface by electrostatic interactions,^[^ ^65^, ^69^, ^70^ ^]^ due to the bacterial negative cell surface charge (zeta potential values of ‐24 mV for *E. coli* and ‐14 mV for *S. aureus*).^[^ ^69^ ^]^ Throughout the test, the chips are illuminated by a collimated broadband light source, and the reflected light is collected and monitored over time. A characteristic reflectance spectrum of the photonic chip (Figure 1A‐iii) exhibits interference fringes from the incident light reflection by the top and the bottom interfaces of the Si microwells (at zero‐order diffraction).^[^ ^7^, ^71^ ^]^ Frequency analysis by fast Fourier transform (FFT) of the reflectance spectrum results in a single peak, where its position along the *x*‐axis corresponds to *2nL, n* representing the refractive index of the filling medium and *L* representing the depth of the microstructures. The amplitude corresponds to the intensity of the reflected light (*I*).^[^ ^66^, ^72^, ^73^ ^]^ Both the *2nL* and *I* values are affected by the bacteria settlement and growth within or on top of the Si microwells and their change over time. These events can be observed in Movie S1 (Supporting Information), depicting the first 25 min following bacteria introduction to the chip. The heterogeneity of the attached cells in terms of cell division is clearly observed, where the cells appear at different stages of cell division. Bacterial cell sizes varied within this observed population and the cell membrane of most of the elongated bacteria began to pinch inward, which is an indicator of cell division through binary fission.^[^ ^74^ ^]^ As such, the *2nL* value increases due to the increase in the refractive index of the filling medium and the intensity decreases due to the light scattering by the bacterial cells.^[^ ^65^, ^66^, ^71^ ^]^ As the intensity responds faster to the presence of bacteria, we focus mainly on *I*, but we will also consider changes in *2nL* values when devising an automatic procedure to predict the susceptibility of a given sample.^[^ ^69^ ^]^ In the presence of an effective antibiotic concentration, bacterial growth diminishes, resulting in a lesser light scattering by the fewer bacterial cells on the surface. This is manifested by a smaller decrease in *I* values compared to bacterial growth without antibiotics, see Figure 1A‐iii. Please note that for the convenience of correlating bacterial growth to a positive signal, we present the changes in *I* values as −Δ*I* throughout this work.

To achieve the goal of carrying out phenotypic AST directly in urine, without prior isolation and processing, our experimental design comprises of three steps as schematically illustrated in Figure 1B. In the first step, we use susceptible bacterial strains to verify in the iPRISM method the antibiotic breakpoint concentrations produced by the CLSI, these values are used to define susceptibility and resistance to the antibacterial agent (Figure 1B‐i). In the second step, by utilizing clinical bacterial isolates, we determine the threshold signal values of the iPRISM method for differentiation between resistant and susceptible strains and correlate our results to those obtained in the clinic using the commercial VITEK 2 AST system (Figure 1B‐ii). Finally, we apply the iPRISM method directly on clinical urine samples and demonstrate its capability for AST of UTI (Figure 1B‐iii). In addition to these steps, we later include a new predictive algorithm to optimize the assay time of the established AST method (Figure 1B‐iv).

### 2.2 Establishing Susceptibility Breakpoints with Clinical Isolates

#### 2.2.1 Susceptibility Determination of Quality Control Strains by iPRISM

The iPRISM method was initially demonstrated for two clinically relevant susceptible bacterial strains, *E. coli* (ATCC 25922) and *S. aureus* (ATCC 29213), which are used by both the CLSI and the EUCAST to validate MIC performance and for quality control in clinical microbiology laboratories.^[^ ^21^, ^22^ ^]^ Moreover, these strains represent both Gram‐negative (*E. coli*) and Gram‐positive (*S. aureus*) bacteria, as well as bacilli and cocci morphologies. The bacterial response to gentamicin, a broad‐spectrum aminoglycoside antibiotic, was investigated to determine the antibiotic breakpoint concentration range in the iPRISM method and its compliance with the values produced by the CLSI. The breakpoint concentrations are used in microbiology laboratory practice to define susceptibility and resistance to antibacterial agents so that the AST results can be interpreted as susceptible, intermediate, or resistant.^[^ ^75^, ^76^ ^]^

**Figure 2:**
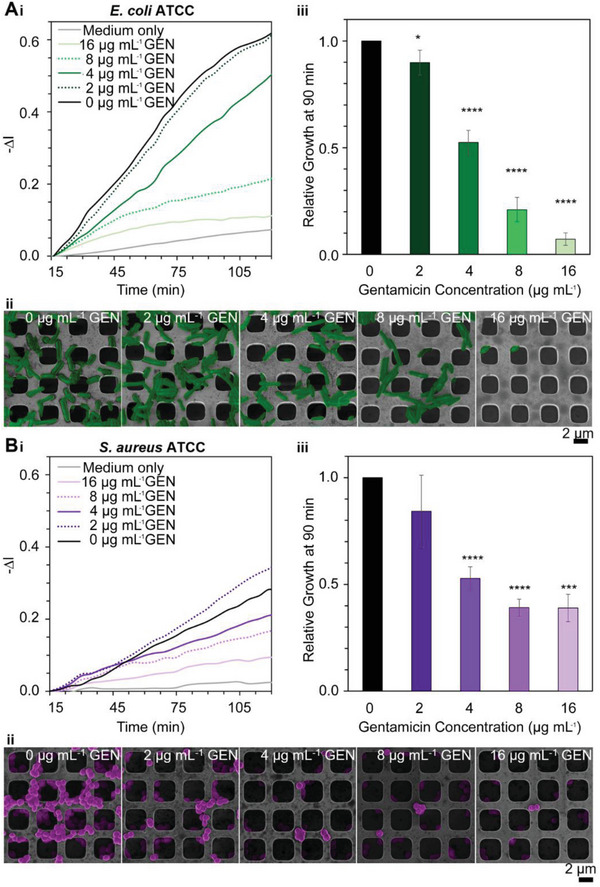
iPRISM AST for A) *E. coli* ATCC 25922 and B) *S. aureus* ATCC 29213 with exposure to different concentrations of gentamicin. i) Representative real‐time iPRISM curves, presenting changes in (−Δ*I*) over time upon exposure of the photonic chips to medium (no bacteria), bacteria (no antibiotic) and bacteria with gentamicin concentrations in the range of 2 – 16 µg mL^−1^. ii) Corresponding HR‐SEM images of the bacteria (false‐colored) at 120 min of the incubation with various concentrations of gentamicin.) iii) Average relative growth values at 90 min after exposure to different gentamicin concentrations (*n* = 20 for *E. coli* ATCC 25922 and n ≥ 6 for S. aureus ATCC 29213, *, *** and **** indicate a statistically significant difference compared to bacteria without antibiotics [0 µg mL^−1^] with *p* < 0.05, *p* < 0.001 and *p* < 0.0001, respectively) of iPRISM AST assay.

Bacterial growth at antibiotic concentrations of 2 to 16 µg mL^‐1^, which are below and above the CLSI standard breakpoints used in the clinic, was monitored in real time for 2 h in a growth medium. The iPRISM signal for both species was observed to gradually increase, as observed when plotting −Δ*I* versus time for each bacterial strain (**Figure** **2**A‐i,B‐i). In the presence of gentamicin concentrations ≥ 4 µg mL^‐1^, the rate of the iPRISM signal increase was lower compared to the bacterial suspension without antibiotics, suggesting a decline in bacterial growth rate due to the bactericidal action of the gentamicin.^[^ ^77^ ^]^

To complement the iPRISM measurements, scanning electron microscopy (SEM) was employed to study the *E. coli* and *S. aureus* populations on the photonic chips following the 2 h incubation at varying gentamicin concentrations, see Figure 2A‐ii,2B‐ii, respectively. Without antibiotics, extensive proliferation is observed for both bacterial species. For *E. coli* (Figure 2A‐ii), a sub‐MIC gentamicin concentration of 2 µg mL^−1[^ ^21^ ^]^ induces some extent of cell aggregation, which can be attributed to the initiation of networks by surface‐associated colonization.^[^ ^32^ ^]^ At higher gentamicin concentrations of 4–8 µg mL^−1^, pronounced filamentation is observed (see also Figure S2A, Supporting Information for a higher magnification SEM image), which indicates the arrest of cell division under stress‐induced antibiotic treatment.^[^ ^33^, ^34^, ^78^ ^]^ Moreover, filamentation has been previously shown to provide an advantage to bacterial populations to form continuous networks by allowing the bridging of non‐adhesive distances of > 5 µm,^[^ ^34^ ^]^ as can be observed in the present case. *S. aureus* also exhibits a distinguishable growth inhibition with increasing antibiotic concentration (Figure 2B‐ii), and a gentamicin concentration of 4 µg mL^−1^ induces morphological anomalies, such as holes (see also Figure S2B, Supporting Information), as was previously observed for other bactericidal agents.^[^ ^79^, ^80^ ^]^ Thus, microwells can provide an advantage in predicting the ability of bacteria to survive on heterogeneous surfaces under antibiotic treatment. At 16 µg mL^−1^ gentamicin concentration, which is above the MIC, the moderate change of iPRISM signal correlates to the few‐remaining cells on the chip surface for both bacteria species, (see corresponding bottom SEM images in Figure 2A‐ii,B‐ii).

The extent of growth inhibition over time for each antibiotic treatment is quantified by normalizing the −Δ*I* values to that of the untreated bacteria, and the results are depicted in Figure S3A,B (Supporting Information) for *E. coli* and *S. aureus*, respectively. For example, after 90 min, the relative growth of *E. coli* exposed to 4 µg mL^−1^ of gentamicin attains a value of 0.7, which corresponds to 30% inhibition, and for 8 µg mL^−1^ the relative growth value is 0.3, i.e., 70% inhibition (see Figure S3A, Supporting Information). While the scale of the relative growth curves ranges between 0 (complete inhibition) to 1 (untreated bacteria), *S. aureus* exposed to a sub‐MIC of gentamicin (2 µg mL^−1^) shows a relative growth >1, corresponding to accelerated growth in comparison to the untreated bacteria (see Figure S3B, Supporting Information). This phenomenon is ascribed to bacterial intrinsic survival strategy, in which sublethal concentrations of antibiotics, or incorrect antibiotic doses, adjust gene expression patterns and metabolism to simultaneously deal with the antibiotic‐induced damage and maintain rapid growth.^[^ ^80^, ^81^ ^]^ The relative growth curves (Figure S3A,B, Supporting Information) stabilize after ≈ 40 min and after this time point, the calculated relative growth values at each studied concentration attain a distinct behavior.

To increase the confidence in the relative growth values, we use the values measured at 90 min, termed as RG_90 min_, to determine the breakpoint gentamicin concentration, and thus the distinction between R and S strains. The mean iPRISM RG_90 min_ values for *E. coli* and *S. aureus*, upon exposure to gentamicin concentrations in the range of 2‐16 µg mL^−1^ are summarized in Figure 2A‐iii,2B‐iii, respectively. The gentamicin standard breakpoints have been established by the CLSI for *E. coli* and *S. aureus* isolates, stating for both strains that MIC values below 4 µg mL^−1^ should be considered as susceptible and those with MIC values >16 µg mL^−1^ should be considered as resistant.^[^ ^21^ ^]^ Likewise, the iPRISM AST demonstrates that at gentamicin concentrations of 4 µg mL^−1^, both bacteria species exhibit a significant growth inhibition of 50%. Thus, using the breakpoint concentration range of 4 µg mL^−1^ can differentiate between gentamicin susceptible and resistant populations within 90 min. These breakpoint values are in excellent agreement with those of traditional AST methods, including disk diffusion tests^[^ ^82^ ^]^ and the VITEK 2.^[^ ^83^ ^]^

To demonstrate the generic nature of the iPRISM assay, we also studied *E. coli* upon exposure to ciprofloxacin (Figure S4A, Supporting Information) and *Enterococcus faecalis* upon exposure to vancomycin (Figure S4B, Supporting Information). We observe a similar trend of a slower increase in the iPRISM signal versus time in the presence of the antibiotics, which is indicative of a decrease in bacterial growth rate. Thus, the iPRISM curves, for all studied bacterial species, enable us to qualitatively distinguish between inhibited and uninhibited bacterial growth in the presence of antibiotics.

#### 2.2.2 Susceptibility Determination Of UTI Bacterial Isolates by iPRISM

**Figure 3:**
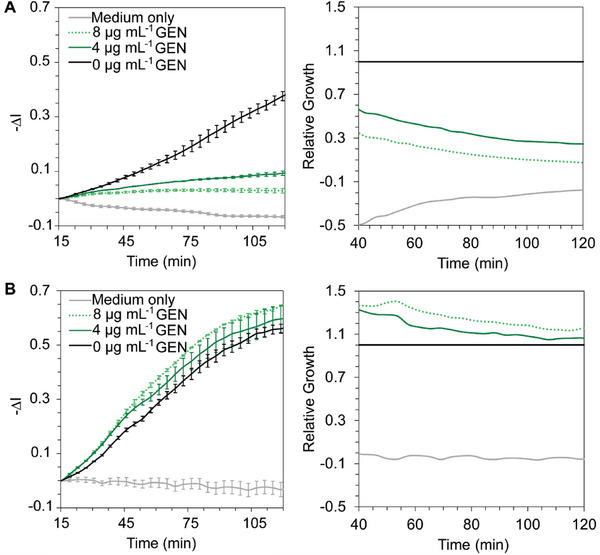
Characteristic iPRISM curves for clinical *E. coli* isolates with exposure to different concentrations of gentamicin. Real‐time iPRISM curves for representative A) susceptible and B) resistant *E. coli* clinical isolates from UTI, upon exposure to different concentrations of gentamicin. The results are presented as −Δ*I* values versus time (left) and the corresponding calculated relative growth values (right) (*n* = 3).

In the next step (Figure 1B‐step ii), to determine iPRISM susceptibility criteria, we observed susceptible and resistant clinical isolates of *E. coli* derived from UTIs (71 in total) and *S. aureus* isolated from BSI (8 in total) against the breakpoint gentamicin concentration range, in the growth medium. Note that the preliminary R/S classification of the isolates was performed at clinical microbiology laboratories using the VITEK 2 system or disk diffusion tests. **Figure** **3** depicts characteristic real‐time iPRISM curves, presenting changes in *‐∆I* versus time, and their corresponding relative growth values, for susceptible and resistant *E. coli* clinical isolates. The susceptible isolate (Figure 3A) depicts significantly inhibited growth upon exposure to both 4 and 8 µg mL^−1^ of gentamicin, with an average relative growth value of 0.3 and 0.1 at 90 min, respectively. For the resistant isolate (Figure 3B), no inhibition is observed upon exposure to 4 and 8 µg mL^−1^ of gentamicin, and the relative growth values are >1. It is interesting to note the enhanced growth rate of the resistant isolate in the presence of gentamicin, emphasizing the clinical implications of treating resistant strains with improper antibiotics. For susceptible and resistant *S. aureus* isolates, similar trends are observed (see Figure S5, Supporting Information); yet the degree of growth inhibition varies between the studied species.

**Figure 4:**
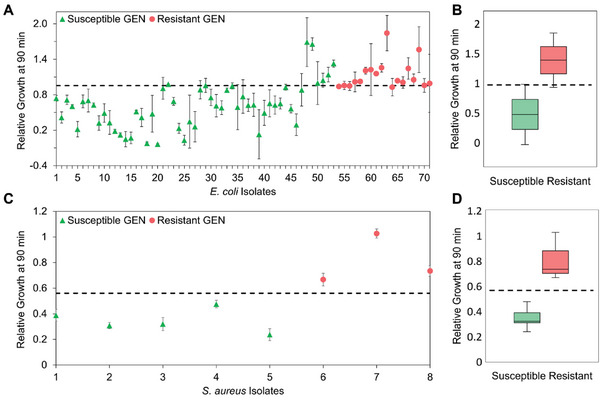
iPRISM phenotypic AST assay establishment for clinical isolates of *E. coli* and *S. aureus*. A) iPRISM relative growth values at 90 min (RG_90 min_) after exposure to gentamicin at a breakpoint concentration of 8 µg mL^−1^ for 53 susceptible and 18 resistant *E. coli* clinical isolates from UTIs (*n* ≥ 2 for each data set) and B) corresponding box plot showing RG_90 min_  =  0.95 as a threshold to differentiate between resistant and susceptible bacteria (please note that 6 false positive isolates were removed from the threshold determination). C) iPRISM relative growth values at 90 min for 5 susceptible and 3 resistant *S. aureus* clinical isolates from bloodstream infections after exposure to 8 µg mL^−1^ gentamicin and D) the corresponding box plot, showing RG_90 min_ = 0.57 as a threshold to differentiate between resistant and susceptible bacteria. The dashed lines indicate the R/S threshold values.

The use of the averaged RG_90 min_ value at the breakpoint concentration of 8 µg mL^−1^ (gentamicin) allows us to differentiate between the R and S isolates with minimal overlap between the two populations, as depicted in **Figure** **4**. For the *E. coli* clinical isolates, we determined the iPRISM RG_90 min_ susceptibility threshold for gentamicin to be 0.95, meaning that *E.  coli* isolates with RG_90 min_ values ≥0.95 will be considered resistant and those with lower RG_90min_ values will be considered as susceptible. This threshold results in high confidence of 92% for *E. coli* in gentamicin R/S determination, with 100% sensitivity and 89% specificity. The specificity was affected by 6/53 false‐positive classification of susceptible *E. coli* strains as resistant. We similarly determined the RG_90 min_ threshold value for *E. coli* clinical isolates upon exposure to ciprofloxacin at a breakpoint concentration of 0.06 µg mL^−1^ (Figure S6, Supporting Information). An RG_90 min_ threshold value of 0.95 results in confidence of 100% in susceptibility determination based on a total of 27 studied clinical isolates. For the clinical isolates of *S. aureus* (Figure 4B), the RG_90 min_ susceptibility to gentamicin threshold is determined to be 0.57, resulting in a confidence level of 100%. Yet, this value may need to be further tuned with the study of more clinical isolates.

These results demonstrate that iPRISM AST can be used to determine susceptibility within 90 min, in comparison to several hours and even days by standard methods.^[^ ^15^, ^16^, ^17^, ^18^, ^19^, ^20^ ^]^ Importantly, the iPRISM method represents the averaged behavior of a population of cells colonizing on the chip, which are free to move, interact and form a community. While there are recent works in which AST is determined faster, they are mainly based on techniques that study the behavior of single cells,^[^ ^30^, ^37^, ^40^, ^56^, ^84^, ^85^, ^86^, ^87^, ^88^, ^89^ ^]^ mostly by imaging.^[^ ^26^, ^27^, ^29^, ^90^, ^91^ ^]^ It is still controversial whether the behavior of immobilized or confined (in a microchannel) single cells is representative of a bacterial population,^[^ ^19^, ^30^, ^32^ ^]^ as it is established that single cells may be more susceptible to bacterial environmental stress in comparison to a community.^[^ ^31^, ^32^ ^]^

### 2.3 Direct AST of Urine using iPRISM

Urinary tract infections are among the most frequent infections encountered in clinical practice worldwide with uropathogenic *E. coli* as the major cause pathogen.^[^ ^92^, ^93^, ^94^ ^]^ UTI is also an example of a medical condition in which rapid AST can provide a better patient outcome by enabling the physician to prescribe the correct antibiotics.^[^ ^95^, ^96^ ^]^ Therefore, we study the feasibility of the iPRISM method to determine bacterial susceptibility directly in urine of UTI patients (Figure 1B, step iii).

First, the iPRISM assay was carried out for urine of healthy asymptomatic volunteers, spiked with *E. coli* ATCC 25922. In a typical experiment, the urine is mixed with growth medium at a 1:1 ratio (with and without antibiotics) and studied directly (no filtration). Figure S7A (Supporting Information) presents characteristic real‐time iPRISM growth curves for *E. coli*‐spiked urine at various gentamicin concentrations, where the growth rate was observed to decrease with increasing gentamicin concentrations. Thus, for the quality assurance strain, the general bacterial behavior in urine is similar to that observed in the growth medium; however, the attained RG_90 min_ values in urine are lower in comparison to those obtained in the rich growth medium, see Figure S7B (Supporting Information). This trend is ascribed to the differences in the composition of the growth medium in the two cases and the ability of urine to sustain growth.^[^ ^97^ ^]^ Yet, a gentamicin breakpoint concentration of 8 µg mL^−1^ is also valid in urine with a degree of inhibition of ∼90%.

**Figure 5:**
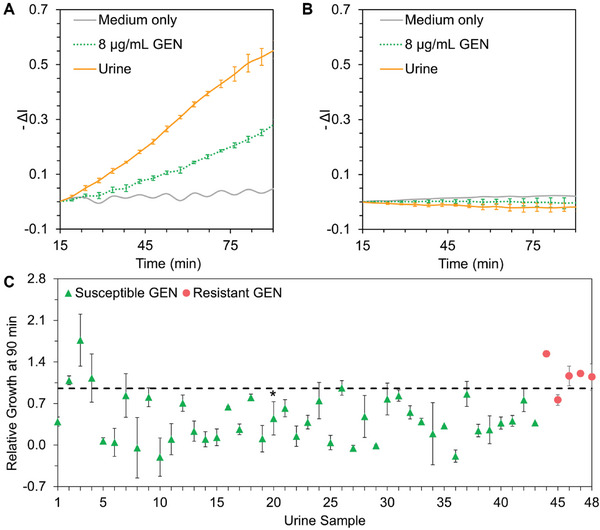
Direct iPRISM AST assay in clinical human urine samples. Representative real‐time iPRISM growth curve of A) positively infected and B) negatively infected clinical urine samples upon exposure to different concentrations of gentamicin. C) iPRISM relative growth values at 90 min after exposure to a gentamicin breakpoint concentration of 8 µg mL^−1^ of urine samples from symptomatic UTI patients. The dashed line indicates the RG_90 min_ = 0.95 threshold value. * for the sample tested for 240 min.

Next, we performed the iPRISM susceptibility study in the urine of symptomatic UTI patients. The demographic characteristics of the UTI patients (age and gender) and the pH value of the urine samples are summarized in Table S1. In these experiments, the collected urine is mixed with the growth medium (at a 1:1 ratio), and studied by iPRISM at the antibiotic breakpoint concentration. **Figure** **5**A,B present real‐time iPRISM growth curves of clinical urine samples positive and negative for UTI, respectively. The iPRISM method allows for the screening of bacteriuria, where a negative urine sample does not show any bacterial growth. Out of 90 collected urine samples (from healthy or symptomatic patients), the iPRISM method determined bacteriuria with 87% sensitivity and 97% specificity at 90 min assay time (**Table** **1**), compared to the conventional culturing method. It should be noted that for a shorter assay time of 30 min, the performance of the iPRISM method is characterized by 82% sensitivity and 94% specificity. Thus, the iPRISM additionally integrates UTI detection into the AST assay with the feasibility of rapid diagnosis.

**Table 1:** Summary of definitive results obtained upon verification by urine culture at the clinical microbiology laboratory and iPRISM analysis (dataset from 90 tested human urine samples at 90 min).

	Urine Culture [clinic]	PRISM Agreement/Disagreement
UTI positive	55	48/7
UTI negative	35	34/1
Total	90	82/8

Figure 5C summarizes the RG_90 min_ values for a total of 48 urine samples, positive for UTI, at a gentamicin breakpoint concentration of 8 µg mL^−1^. The results show that we can successfully differentiate between susceptible and resistant infections using the established threshold of RG_90 min_  = 0.95, where the resistant urine sample presents RG_90 min_ values greater than 0.95 and the susceptible samples present RG_90 min_ values below this threshold. Thus, these experiments demonstrate the feasibility of iPRISM to determine bacterial susceptibility directly in urine. The overall performance of the iPRISM AST assay for gentamicin susceptibility of UTIs is 80% sensitivity and 91% specificity, compared to the VITEK 2 results.

Additionally, 24 clinical urine samples were studied against ciprofloxacin at a breakpoint concentration of 0.06 µg mL^−1^ (see Figure S8, Supporting Information), showing a sensitivity of 100% and a specificity of 94%. For both tested antibiotics, higher than 90% categorical agreement following CLSI guides was obtained for AST (92% in gentamicin case and 96% for ciprofloxacin case, respectively).^[^ ^98^ ^]^ Thus, the iPRISM shows immense potential for direct and rapid susceptibility determination of UTI, where we skip the laborious lengthy steps of plating, overnight incubation, and isolation, which encompass the traditional workflow in clinical laboratories, and greatly accelerate the subsequent AST step in comparison to the practiced state‐of‐the‐art methods. Based on these preliminary studies, the iPRISM assay can potentially provide a realistic time to result of <90 min. Yet, additional experiments are needed to establish this ambitious workflow for variations between samples.

It should be emphasized that in all our studies raw and unprocessed urine samples were used. Hence, the urine variable intrinsic optical density as well as the presence of other bacterial contaminations (typically in the range of hundreds of cells mL^−1^) does not affect the iPRISM results (see the raw sample denoted as “Urine” in Figure S6A, Supporting Information). The −Δ*I* signal of this sample remains unchanged at the first 2 h of the assay with no sign of the proliferation of other bacteria originating from the raw urine. Thus, the iPRISM allows for direct processing of urine samples without prior processing, such as separation and pre‐concentration of the pathogen, or removal of large particles (via filtration or centrifugation), which are practiced in emerging AST techniques.^[^ ^29^, ^37^, ^52^, ^61^, ^99^ ^]^ The pre‐processing steps may significantly delay the test due to the large scale of sample handling in a routine workload of clinical laboratories.

### 2.4 Accelerating iPRISM AST by Machine Learning

The iPRISM assay is capable of generating characteristic measurements that can be used to differentiate between resistant and susceptible bacteria (Figure 3). Although iPRISM signals are informative enough that AST can be performed through visual inspection, we developed a predictive model that can standardize and accelerate the assay's discrimination time in an automatic manner, and most importantly predict bacterial susceptibility in real‐time. Our machine learning model is comprised of a regularized logistic regression model operating on features obtained via a Dynamic Time Warping (DTW) kernel.^[^ ^100^ ^]^ DTW provides a notion of distance measurement amenable to comparing time series and that is popular in machine learning and signal analysis.^[^ ^101^, ^102^, ^103^ ^]^ DTW is both robust and flexible; it is invariant to time shifts and can be used to compare signals of different lengths. These characteristics enable our model to characterize bacterial growth irrespective of the lag phase duration and be easily applied to iPRISM measurements sampled at any frequency. By applying DTW to compare iPRISM measurements from unknown samples with those in our training set, we can obtain a sense of similarity that is informative of the susceptibility of new samples. In particular, we use both intensity and *2nL* signals from the assay, since while the intensity responds faster to the presence of bacteria, *2nL* provides complementary information that can also be leveraged to provide a more accurate prediction.

**Figure 6:**
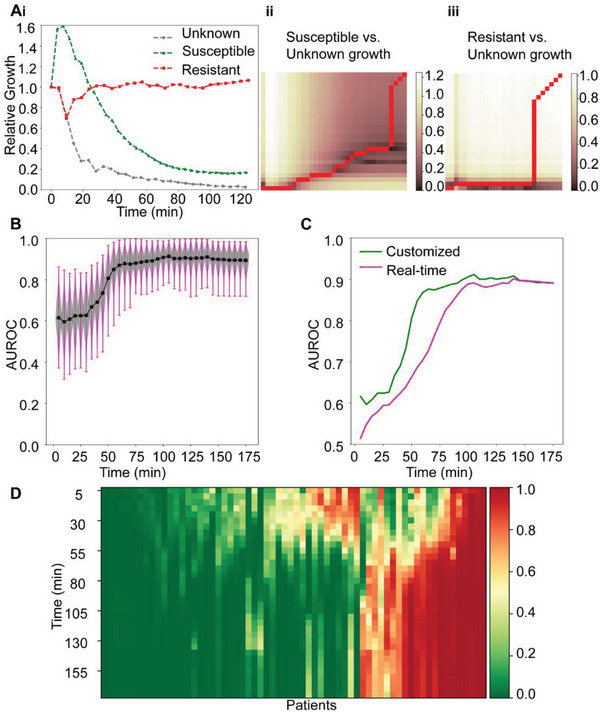
iPRISM AST machine learning based on clinical *E. coli* isolates upon exposure to gentamicin. A) Dynamic Time Warping on iPRISM relative growth curves. i) Relative growth curves of Susceptible, Resistant, and Unknown bacterial strains. ii) Cost matrix showing the pairwise distance between every point in the Unknown and Susceptible curves. The optimal warping path is shown in red. iii) Similarly, the cost matrix and optimal warping paths for the Unknown and Resistant curves. Note that each of the signals compared are sampled at different frequencies. B) Customized model AUC performance over 1000 bootstraps. C) Comparison between the customized and real‐time models. The performance gap between the optimal and practical models is generally small, and negligible after 100 min. Note that the performance of both configurations plateaus at an AUC of 0.9 after 90 min, indicating the stable point of the model. D) Prediction stability. Model predictions were assessed per patient as a function of time, with 0.0 indicating strong confidence in a susceptible strain, and 1.0 strong confidence in a resistant strain. We can clearly see that for the majority of samples, after 90 min the predictions are constant.

As a brief demonstration, we compared the relative growth curves between a resistant, a susceptible, and an unknown bacterial strain. Note that each curve was sampled at a different frequency (**Figure** **6**A‐i). The lack of uniform sampling makes it difficult to quantify the distances between the three curves since we cannot simply compare the relative growth at each time point without interpolation. Intuitively, however, we can see that the Unknown growth curve is closer to the Susceptible rather than the Resistant curve. DTW resolves this issue by finding the optimal alignment between two time series, allowing for the similarity between distinctive patterns to be quantified independently of their sampling and length. Mathematically, this can be expressed as computing the distance between every point in the two curves, and then finding a warping path, or transformation, that minimizes this cost subject to specific constraints (Equation S1, Supporting Information). This warping path is akin to synchronizing the two curves, and the DTW distance is simply the distance obtained for the most optimal synchronization. The optimal warping path aligning the Susceptible and Unknown strains is shown in Figure 6A‐ii, and produces a distance of 1.12. In comparison, the optimal warping path aligning the Resistant and Unknown strains (Figure 6A‐iii) produces a distance of 4.36. The distance between the Susceptible curve to the Unknown curve is shorter than the distance from the Resistant curve to the Unknown curve, which confirms the utility of DTW since the Unknown curve is actually of susceptible origin.

We can further improve the predictive power of DTW with machine learning. Given a set of labeled iPRISM curves, we can predict the identity of a new sample at time *t* as follows:

(1) $$\hat{y}=\mathrm{sgn}\left[\sum_{i\;=\;1}^{n}w_{i}y_{i}DTW\left(x_{i}\left(t\right),\;x^{\prime }\right)\right]$$

where (*x_i_* (*t*),*y_i_*) for *i* ∈ [1, *n*] indicates a training set of *n* labeled multivariate iPRISM signals truncated to time *t*, $\left(x^{\prime },\hat{y}\right)$ denote the unlabeled iPRISM curve and predicted label respectively, and *w_i_*, *i* ∈ (1, *n*) are the learned weights of the model. In particular, the iPRISM signals of interest measure the intensity and frequency shift at different antibiotic concentrations. The magnitude of *w_i_* indicates the contribution each labeled sample has on the prediction, allowing for our model to account for the variability between samples rather than simply (uniformly) averaging over the *DTW* distances. In the course of training, this model can learn to decrease the influence of a particularly noisy sample, while increasing that of a more reliable class exemplar.

Our model is simple to train and can be instantiated for a specific time, *T*, or as a real‐time predictor, for any time *T*, as we now explain. If the desired inference or target time is known in advance, our model can be optimized to predict the susceptibility of iPRISM signals up to that specific time. While less flexible (having to specify the read‐out time in advance), these customized models offer the optimal performance for predicting at that specific time. In contrast, if the user desires to infer bacterial susceptibility in real‐time (for any time) a single real‐time model can be trained to continuously update its prediction as new iPRISM measurements are acquired. The performance of the real‐time model configuration offers maximum flexibility while sacrificing minimal performance.

We benchmark the performance of both modes using Leave‐One‐Out evaluation across 65 clinical *E. coli* isolates upon exposure to gentamicin (the visual inspection of these samples is shown in Figure 4A), and report the distribution of the Area Under the Receiver Operating Characteristic Curve (AUC) scores across 1000 bootstraps samples. Our experiments demonstrate that customized models achieve a mean AUC of 0.9 after only 60 min of iPRISM measurements, and the real‐time model after 90 min (Figure 6B,C). Beyond predictive accuracy, we carefully evaluate and examine model stability. A model is said to be stable if there exists a point in time (the stable point) after which the additional measurements will no longer alter the predicted classification. This is of practical relevance to the AST problem because it provides a threshold in time after which users can be confident in the predicted susceptibility of a new bacterial sample. We observe that under both configurations, our model achieves stability after 90 min of iPRISM measurements (Figure 6C,D) due to the convergence of the underlying DTW distances (Figure S9, Supporting Information). Such observations come into agreement with the visual inspection of iPRISM AST results as described in the previous session.

Overall, our algorithm is both powerful and intuitive and operates under the natural assumption that iPRISM curves of a resistant sample are more similar to those of other resistant samples, rather than the iPRISM measurements of susceptible samples and vice versa. However, instead of relying on visual inspection to determine “similarity”, we are able to precisely quantify this notion using DTW. Furthermore, by training a classifier over many samples and multivariate signals, we are able to automatically provide robust, stable predictions.

## 3 Conclusion

In summary, we have established the iPRISM assay for a rapid AST of clinical UTI urine samples.

The assay exploits the interface between Si microstructured topologies and colonized bacteria in suspension and allows for real‐time and label‐free monitoring of bacteria growth and its response to antibiotic agents, without the use of elaborate microscopy or sophisticated equipment. The versatility of the assay is demonstrated by the analysis of different bacterial strains, which were clinically isolated from UTI or BSI, and different antibiotic agents. Importantly, the assay has been established for analysis of unprocessed urine specimens of UTI patients and bacterial antibiotic susceptibility determination has been achieved within 90 min through an experimental analysis of a large dataset of clinical samples and via a machine learning algorithm. The iPRISM assay thus profoundly accelerates AST results in comparison to conventional manual methods and state‐of‐the‐art automated AST systems, routinely used in clinical laboratories.^[^ ^17^ ^]^

The iPRISM assay monitors a large population of thousands of cells, which colonize on the chip and are free to move, interact and form a community. As such, the assay is less prone to both false‐negative and false‐positive readouts, ascribed to differences in endogenous stress‐resistance levels between individual cells in the same bacterial population.^[^ ^104^ ^]^ Yet, similar to other phenotypic‐based methods for rapid AST, we speculate that time‐to‐results may vary with a combination of bacterial species and antibiotics including doubling time of different bacterial species, and the antibiotic‐induced mechanism.^[^ ^105^, ^106^, ^107^ ^]^ Thus, attention is required to handle polymicrobial urine specimens by iPRISM AST, in which multi‐pathogens with heterogeneous antibiotics response coexist. Our future efforts are directed to the generalization of the iPRISM AST and its establishment for other sample types, aiming to simplify sample preparation techniques, bacterial cell loads and diminish background signals of complex biological samples. In terms of potential point‐of‐care compatibility of the iPRISM assay, the photonic sensors are integrated in multiplexed disposable microfluidic devices, which can be easily adapted and produced on a large scale, requiring low sample and reagent volumes, and has such holding promise to improve current UTI management at potentially lower costs.

## 4 Experimental Section

### Materials

Brain heart infusion (BHI) broth and Bacto agar were supplied by Difco, USA. Gentamicin sulfate, ciprofloxacin, vancomycin, glutaraldehyde, and *N*’ (3‐triimethoxysilylpropyl) diethylenetriamine (APTES) were purchased from Sigma‐Aldrich, Israel. Tryptic soy agar (TSA) supplemented with 5% defibrinated sheep blood agar plates was purchased from Novamed Ltd., Israel. Absolute ethanol and all phosphate‐buffered saline (PBS) salts were supplied by Merck, Germany. Acetone was supplied by Gadot, Israel. Acetic acid and glycerol were supplied by Bio‐Lab Ltd., Israel. Photoresist AZ4533 was supplied by Metal Chem Ltd., Israel.

### Preparation of Solutions and Media

All aqueous solutions were prepared in Milli‐Q water (18.2 MΩ cm). BHI medium (37 g L^−1^) was prepared according to the manufacturer's instructions. BHI agar plates were prepared from BHI medium and bacto agar (18 g L^−1^). PBS was constituted of NaCl (137 mm), KCl (2.7 mm), KH_2_PO_4_ (1.8 mm), and Na_2_HPO_4_ (10 mm). All solutions and media were autoclaved or sterile‐filtered prior to use.

### Bacterial Strains and Clinical Samples

*E. coli* ATCC 25922, *S. aureus* ATCC 29213, and *Enterococcus faecalis*  ATCC 29212 from American Type Culture Collection (ATCC) were purchased from Biological Industries, Israel.

Clinical isolates from patients with urinary tract infections (UTI) and bloodstream infections (BSI) were obtained from the clinical microbiology laboratories of HaEmek Medical Center and Bnai Zion Medical Center, Israel. Resistant and susceptible determinations of the clinical isolates were performed by the hospital clinical microbiology laboratory using the VITEK 2 automated system (bioMérieux, France) and the disk diffusion test.

All bacterial strains were grown overnight on BHI agar plates or TSA plates at 37 °C and then inoculated in liquid BHI overnight at 37 °C. The overnight‐grown liquid culture was mixed with 50% glycerol at a volume ratio of 1:1 and stored at −80 °C until further use.

Anonymous discarded urine samples were obtained from Bnai Zion Medical Center with the consent of Bnai Zion IRB. Due to safety regulations during the COVID‐19 pandemic, the urine samples were kept refrigerated for at least 2 days until the clinical AST results were obtained. Ethical approvals BNZ 0110–14.

### Fabrication of iPRISM Cassettes

Si photonic chips with a periodic porous microstructure were fabricated using standard lithography and reactive ion etching techniques at the Micro‐Nano‐ Fabrication and Printing Unit at the Technion ‐ Israel Institute of Technology. The microstructured Si samples consisted of an array of square wells, with a width of ∼2.7 µm, depth of 4 µm, and ∼0.7 µm thick walls. The samples were oxidized for 0.5 h at 850 °C and pre‐coated with a photoresist to protect the delicate microstructure during the subsequent dicing process (automated dicing saw, DAD3350 Disco, Japan) in which 4 × 4 mm chips were obtained. The latter were cleaned with acetone and allowed to react with 2% v/v APTES solution for 1 h, after which the samples were thoroughly rinsed with ethanol. The resulting photonic chips were integrated into 7.6 cm × 2.5 cm acrylic microfluidic cassettes constructed by Potomac Photonics, Inc. (Maryland, USA). Each device contains ten integrated photonic chips with microchannels. Devices are fabricated from four laser‐cut polymethyl methacrylate layers laminated by pressure‐sensitive adhesive: a plain base layer, a layer containing square cut‐outs for the individual chips, layer containing the liquid channels resulting in 40 µL volume, and a top sealing layer containing a syringe‐compatible injection port and outlet port. These iPRISM devices were sterilized by ethylene oxide vapor before use.

### Optical Setup, Data Acquisition, and Analysis

A bifurcated fiber optic (Ocean Optics, USA) implemented with a collimating lens was positioned normally to the iPRISM cassette; the latter was positioned on a motorized linear stage (Thorlabs Inc., USA) heated to 37 °C. A tungsten light source was used for illumination, and reflectivity spectra were collected perpendicular to the surface at a wavelength range of 450−900 nm by a charge‐coupled device (CCD) spectrometer (USB4000, Ocean Optics, USA). The stage movement and spectral measurements were controlled by LabView software (National Instruments, USA).

Frequency analysis of the spectra was performed using Matlab Software R2020b. The resulting peak after a fast Fourier transform (FFT) was identified by determining the maximum peak position where the height of the detected peak corresponds directly to the intensity of the reflected light. The changes in the intensity peak values (termed as *ΔI*) over time were calculated as follows:

(2) $$\Delta I=\frac{I-I_{15}}{I_{15}}$$

in which *I*_15_ represents the intensity reading collected after an initial 15‐min incubation of the chip with the respective studied samples within the iPRISM cassette. This short conditioning time allowed the bacteria to settle within the Si microstructures.^[^ ^65^ ^]^

### AST Assay

Bacteria were grown overnight on TSA plates (supplemented with 5% defibrinated sheep blood agar) at 37 °C, and formed colonies were transferred to BHI media and incubated for 0.5 h. Subsequently, bacteria suspensions were diluted to a final turbidity of 0.5 McFarland standard, according to the CLSI guidelines (which corresponds to an optical density value at 600 nm, OD_600_, of 0.1 in our studies). The resulting neat suspensions or pre‐mixed with a gentamicin solution (at a total volume of 40 µL) were injected into each channel of the iPRISM device. Note that gentamicin concentrations were chosen according to CLSI breakpoint concentrations (2, 4, 8, 16 µg mL^−1^). Then, the inlet and outlet ports of the iPRISM device were sealed with a Breath‐Easy sealing membrane (Z380059; Sigma‐Aldrich). The reflectance signal was collected and analyzed throughout the experiment for at least 90 min, using the optical setup described in the previous section.

For urine AST testing, refrigerated urine samples were mixed with BHI medium at a volume ratio of 1:1 and incubated for 1 h at 37 °C in order to revive their growth, achieving a final turbidity of 0.5 McFarland. Next, gentamicin was added, and the experiment was initiated. For the preliminary spiked urine studies, fresh urine samples donated by healthy asymptomatic volunteers were directly spiked with *E. coli* ATCC 25922 or *E. coli* clinical isolates under the described conditions.

### Electron Microscopy Studies

Focused ion beam cross‐sectional SEM was performed using a Dual Beam Helios NanoLab G3 instrument (FEI, USA). Platinum deposition was conducted prior to observation. High‐resolution scanning electron microscopy (HR‐SEM) was carried out using a Zeiss Ultra Plus high‐resolution scanning electron microscope equipped with a Schottky field‐emission gun (Carl Zeiss, Germany) operating at an acceleration voltage of 1 kV. For HR‐SEM imaging, sample fixation was performed at the end of the 2 h growth assays with 2.5% glutaraldehyde, followed by overnight soaking in PBS at 4 °C. Then a series of dehydration steps were conducted in 10%, 25%, 50%, 75%, and 100% ethanol solutions (20 min in each solution). Selected HR‐SEM images were false‐colored using Adobe Photoshop software CS3.

### Machine Learning and Dynamic Time Warping

Open‐source Python libraries were used to implement the machine‐learning algorithms. Data cleaning and preprocessing were performed by *pandas* version 1.3.5, and the regularized logistic regression classifier was trained using *sklearn* version 1.0.2 and *numpy* 1.21.6. Dynamic time warping distances were computed using the *tslearn* library version 0.5.2. Plots and figures were generated with *matplotlib* version 3.5.2.

### Statistical Analysis

The statistical significance of the treatment groups was evaluated using Student's t‐test. *p < 0.05, ***p < 0.001, and ****p < 0.0001 were considered statistically significant in analyses. Statistical details of the experiments were included in the figure captions.

## Conflict of Interest

The authors declare no conflict of interest.

## Supporting information

Supporting Information

Supplemental Movie 1

## Data Availability

The data that support the findings of this study are available from the corresponding author upon reasonable request.
