# Supplementary material for: Accurate Prediction of Antimicrobial Susceptibility for Point‐of‐Care Testing of Urine in Less than 90 Minutes via iPRISM Cassettes
Source: Adv Sci (Weinh). 2023 Aug 16;10(31):2303285. doi: 10.1002/advs.202303285 (PMC10625094; doi:10.1002/advs.202303285)
Supplement: Supplementary file 1 — Supporting Information [file ADVS-10-2303285-s002.pdf]

ADVANCED SCIENCE
Open Access

Supporting Information

for *Adv. Sci.*, DOI 10.1002/advs.202303285

Accurate Prediction of Antimicrobial Susceptibility for Point-of-Care Testing of Urine in Less than 90 Minutes via iPRISM Cassettes

Xin Jiang, Talya Borkum, Sagi Shprits, Joseph Boen, Sofia Arshavsky-Graham, Baruch Rofman, Merav Strauss, Raul Colodner, Jeremias Sulam, Sarel Halachmi, Heidi Leonard and Ester Segal**

Supporting Information

Accurate Prediction of Antimicrobial Susceptibility for Point-of-Care Testing of Urine in Less than 90 Minutes via iPRISM Cassettes

Xin Jiang, Talya Borkum, Sagi Shprits, Joseph Boen, Sofia Arshavsky-Graham, Baruch Rofman, Merav Strauss, Raul Colodner, Jeremias Sulam, Sarel Halachmi, Heidi Leonard, and Ester Segal**

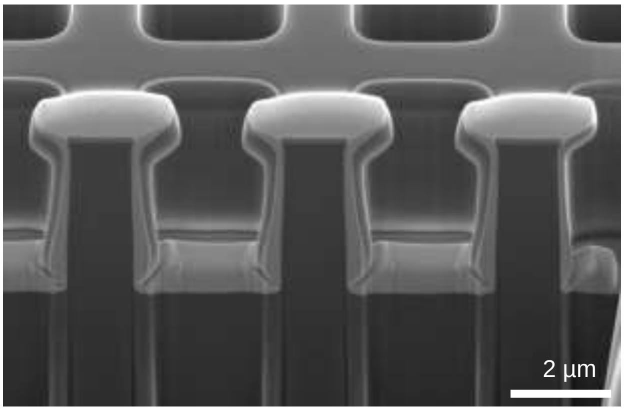

Figure S1. Representative cross-sectioned focused ion beam-scanning electron micrograph of the silicon microwells.

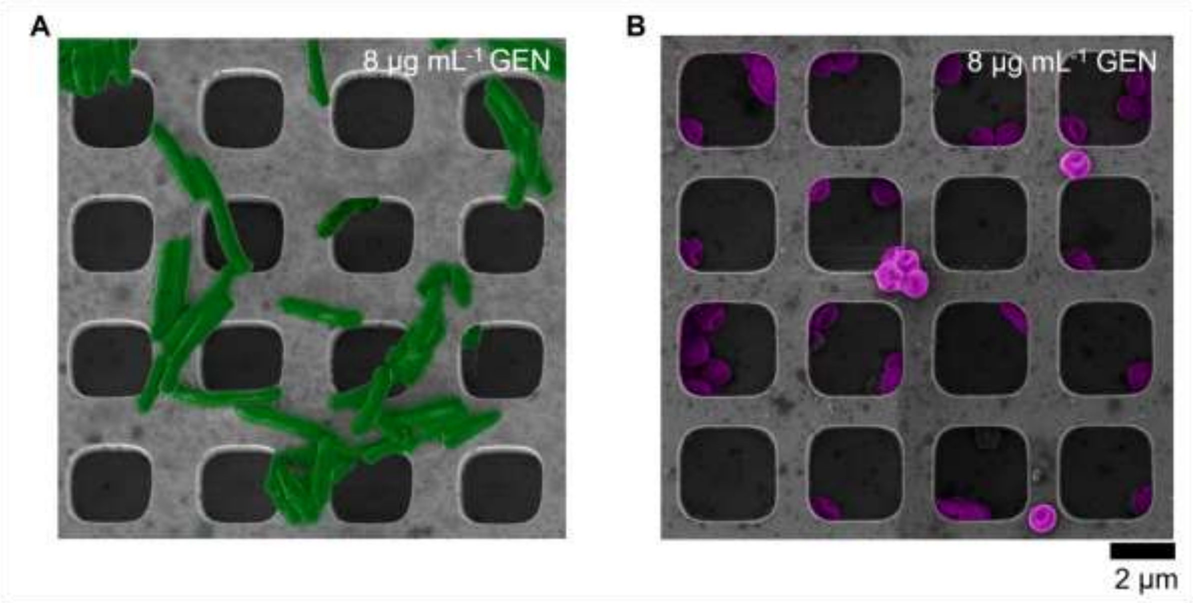

Figure S2. HR-SEM images of A) *E. coli* ATCC 25922, B) *S. aureus* ATCC 29213 after incubation with 8 µg mL^{-1} gentamicin, demonstrating filamentation of *E. coli* cells and damaged *S. aureus* cells (with holes).

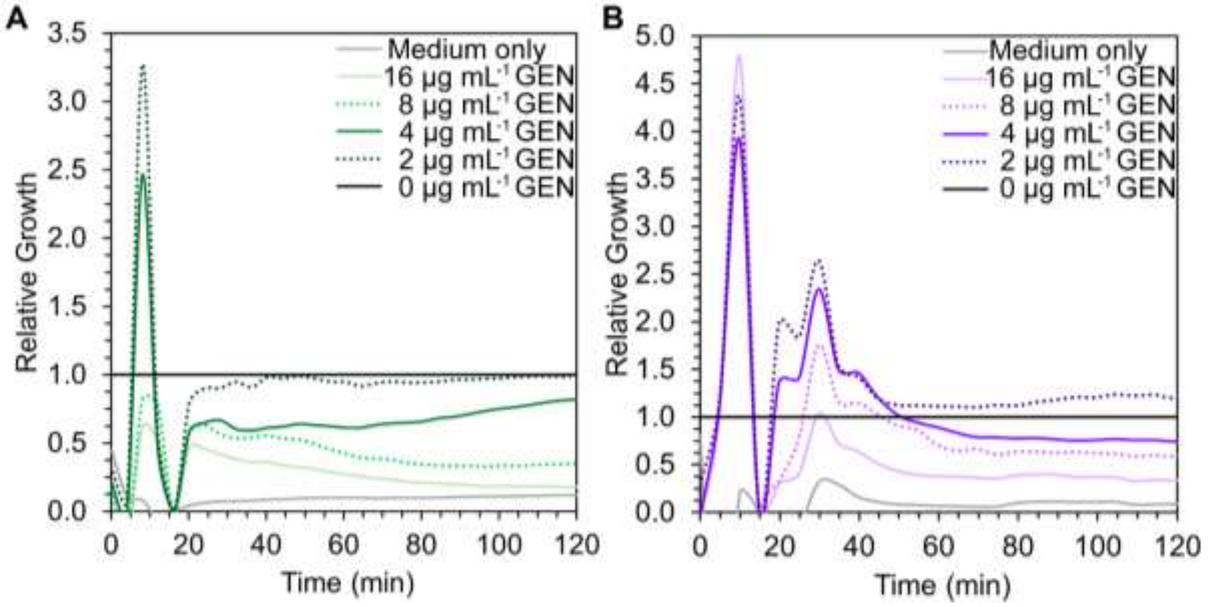

Figure S3. iPRISM relative growth vs. time curves corresponding upon exposure to different concentrations of Gentamicin, calculated as the fraction of bacteria growth compared to the growth without antibiotics for A) *E. coli* ATCC 25922 and B) *S. aureus* ATCC 29213.

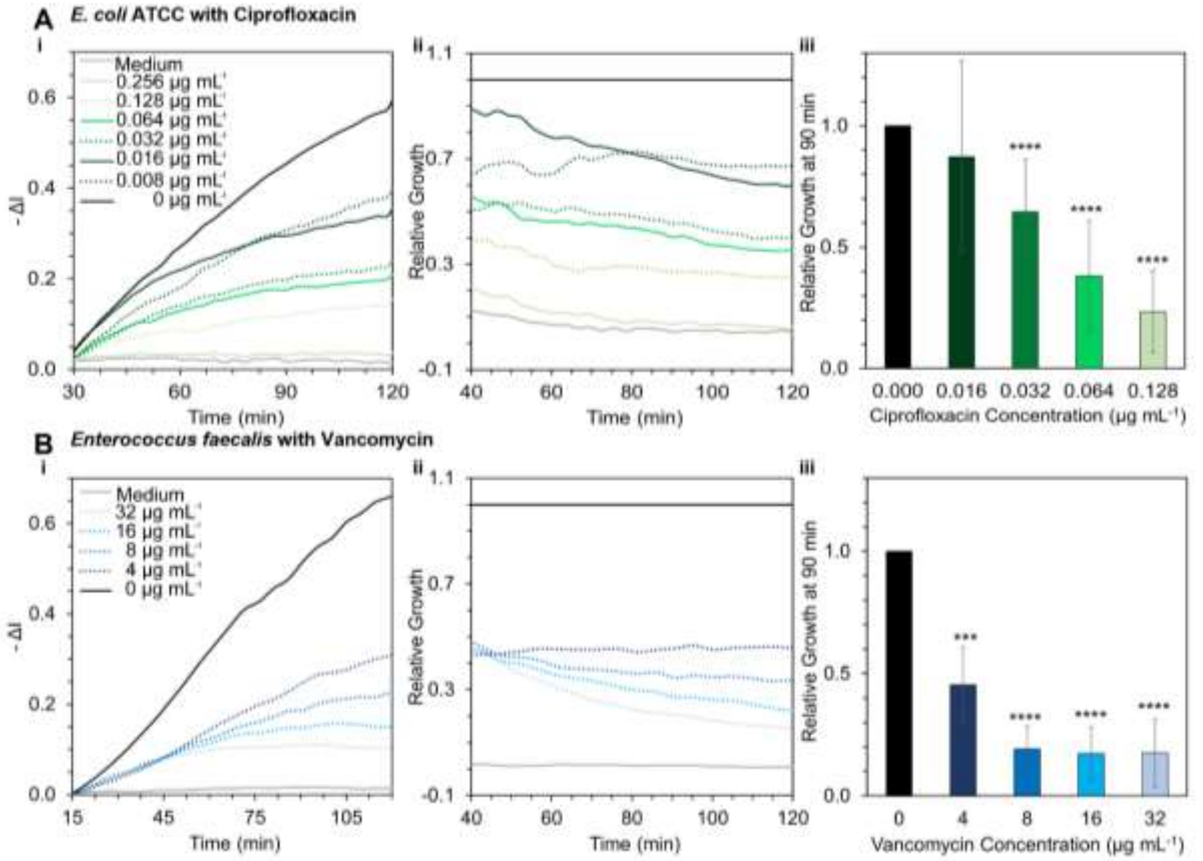

Figure S4. iPRISM AST assay for A) *E. coli* ATCC 25922 upon exposure to different concentrations of ciprofloxacin and B) *Enterococcus faecalis* upon exposure to different concentrations of vancomycin. i) Representative real-time iPRISM curves, depicting changes in (-ΔI) over time upon exposure of the Si microstructures to medium (no bacteria), bacteria (no antibiotics) and bacteria with increasing antibiotic concentrations. ii) Corresponding relative growth over time, calculated as the fraction of bacteria growth compared to the growth without antibiotics. iii) Averaged relative growth values at 90 min after exposure to different antibiotic concentrations ($n \geq 6$, *** and **** indicate a statistically significant difference compared to bacteria without antibiotics [0 µg mL^{-1}] with $p < 0.001$ and $p < 0.0001$, respectively).

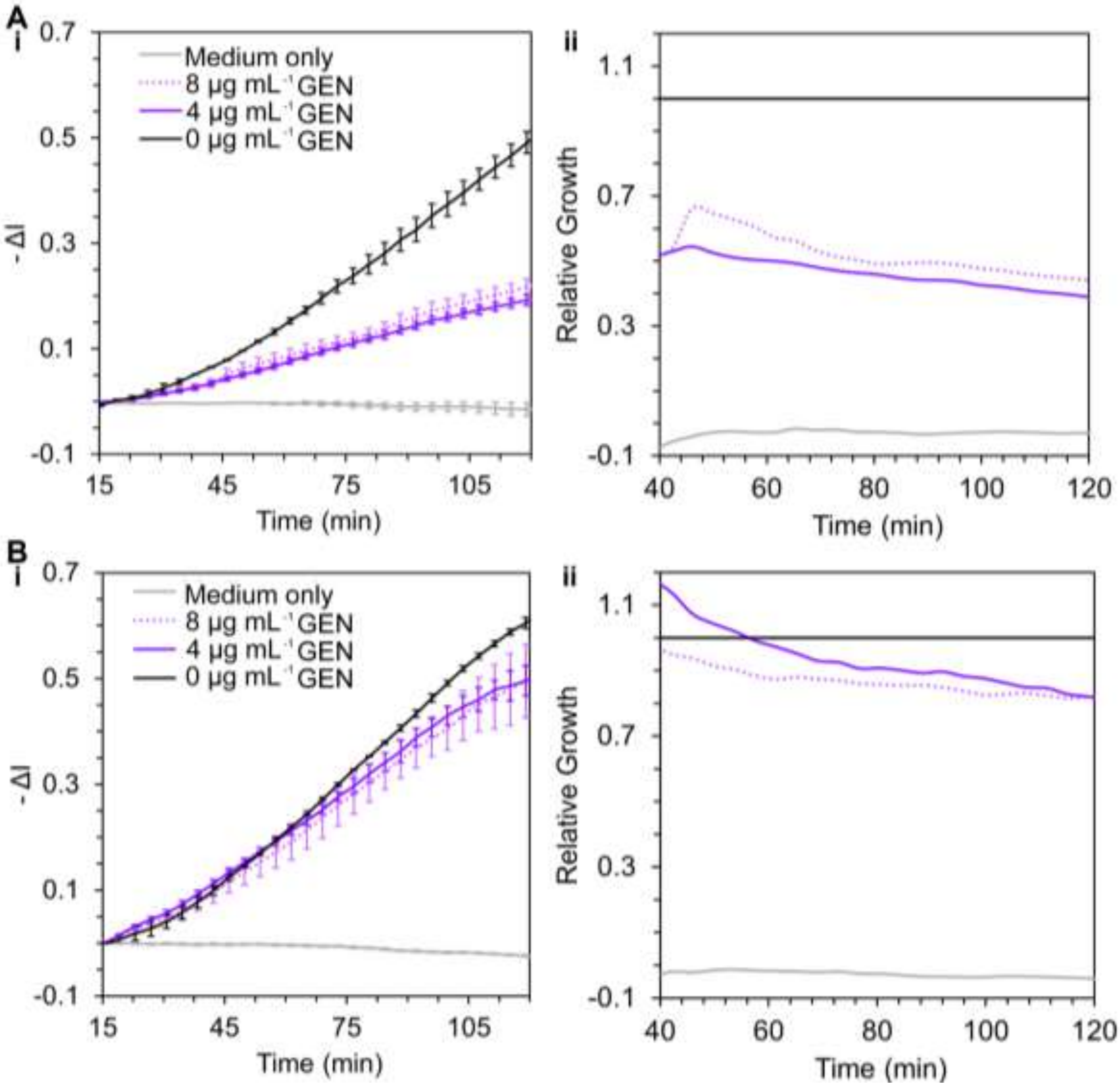

Figure S5. Real-time iPRISM curves for A) susceptible and B) resistant *S. aureus* clinical isolates from BSI upon exposure to different concentrations of gentamicin. The results are presented as -ΔI values vs. time (left) and the corresponding calculated relative growth values (right) (n=3).

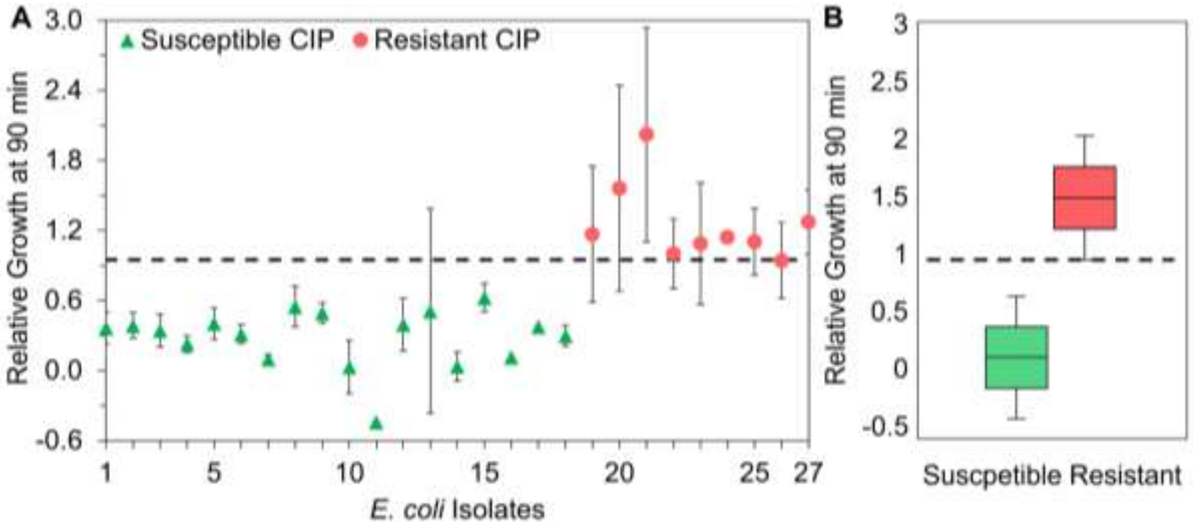

Figure S6. iPRISM AST assay demonstration for *E. coli* upon exposure to ciprofloxacin breakpoint concentration of 0.06 µg mL^{-1}. A) iPRISM relative growth values at 90 min ($RG_{90\ min}$) for 27 susceptible or resistant *E. coli* clinical isolates from UTIs ($n \geq 2$ for each data set) and B) corresponding box plot showing $RG_{90\ min}$= 0.95 as a threshold to differentiate between resistant and susceptible bacteria.

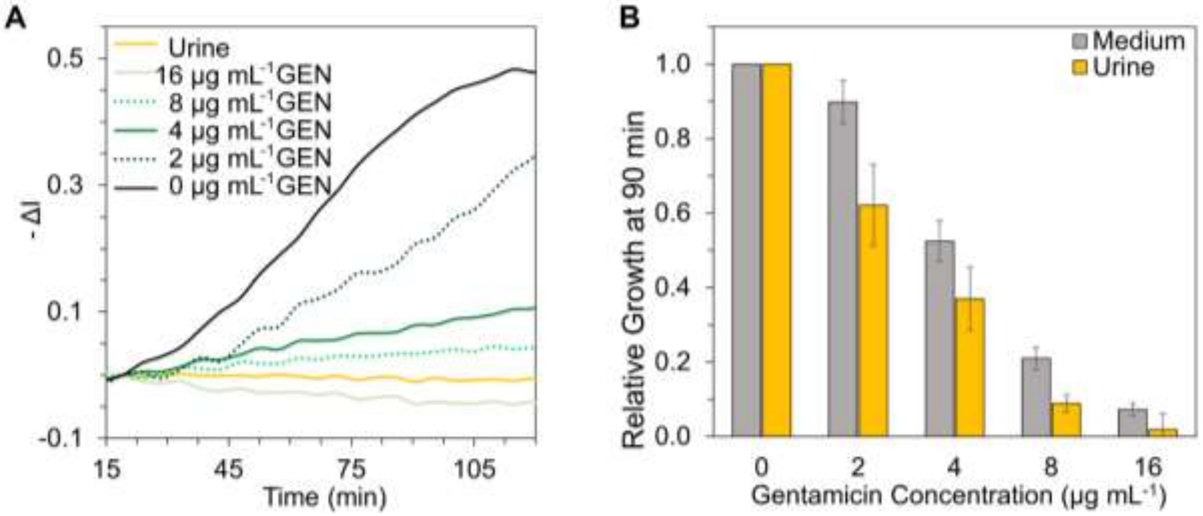

Figure S7. Direct iPRISM AST assay in clinical human urine samples. A) Representative real-time iPRISM growth curve of urine sample spiked with *E. coli* ATCC 25922, upon exposure to different concentrations of gentamicin. B) Comparison of the iPRISM relative growth values at 90 min ($RG_{90\ min}$) values for *E. coli* ATCC 25922 spiked in growth medium or in urine sample, upon exposure to different concentrations of gentamicin ($n \geq 3$ for each data set).

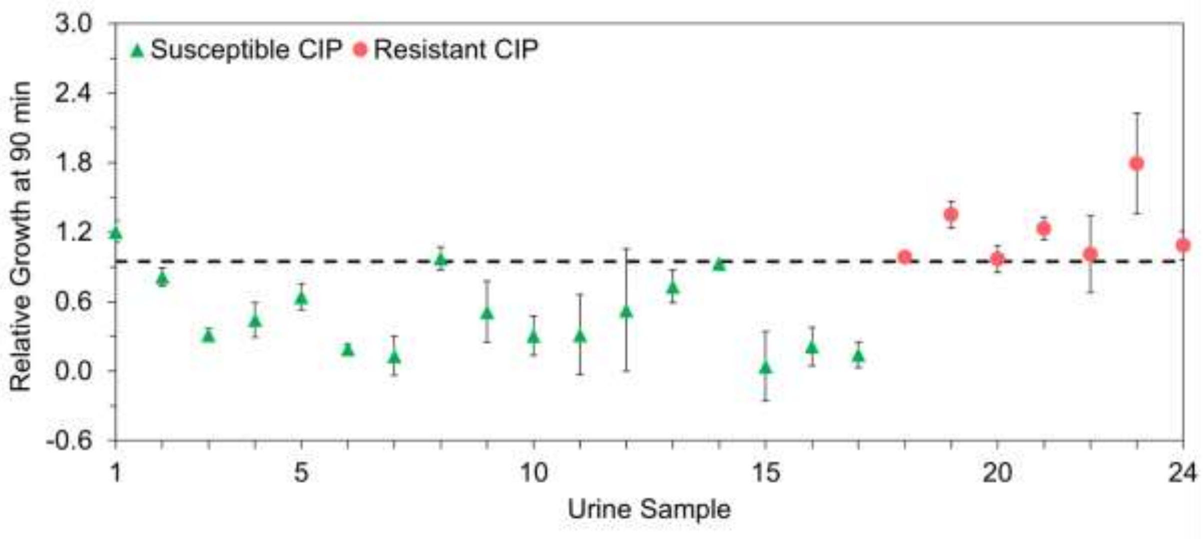

Figure S8. Direct iPRISM AST assay in clinical human urine samples for *E. coli* upon exposure to ciprofloxacin breakpoint concentration of 0.06 µg mL^{-1}. iPRISM relative growth values at 90 min. Dashed lines indicate the $RG_{90\ min}$= 0.95 threshold value.

Supplementary note 1: Dynamic Time Warping Calculation

Formally, given two signals P and Q of length m and n, respectively, the dynamic time warping distance between them DTW(P,Q) is given as follows: [97]

$$\mathrm{DTW}(P,Q) = \min_{\pi \in A(P,Q)} \sqrt{\sum_{(i,j)\in\pi} \|P_i - Q_j\|} \tag{S1}$$

Where the minimization is performed over all valid alignment paths π. An alignment path π of length K is a sequence of K index pairs $((i_0, j_0), \ldots\ (i_K, j_K))$ and is admissible if and only if it satisfies the following conditions.

Boundary: $\pi_0 = (0{,}0)$ and $\pi_K = (n-1, m-1)$

Monotonicity: $i_0 \leq i_i \leq \ldots \leq i_K$ and $j_0 \leq j_1 \leq \ldots \leq j_K$

Step size: $(i_{l+1} - i_l, j_{l+1} - j_l) \in [(0{,}0), (0{,}1), (1{,}0), (1{,}1)]\ for\ 0 \leq l \leq K-1$

Intuitively, if we construct a cost matrix C, where $C_{(i,j)} = \|P_i - Q_j\|$, then the optimal alignment path is the “valley” of lowest cost running from the bottom left to the top right of C. The boundary condition enforces that the starts at the bottom left corner and ends at the top right corner, the monotonicity condition ensures that does not ever turn “backwards” or cross over itself, and the step size ensures that no “jumps” are made (Figure 6).

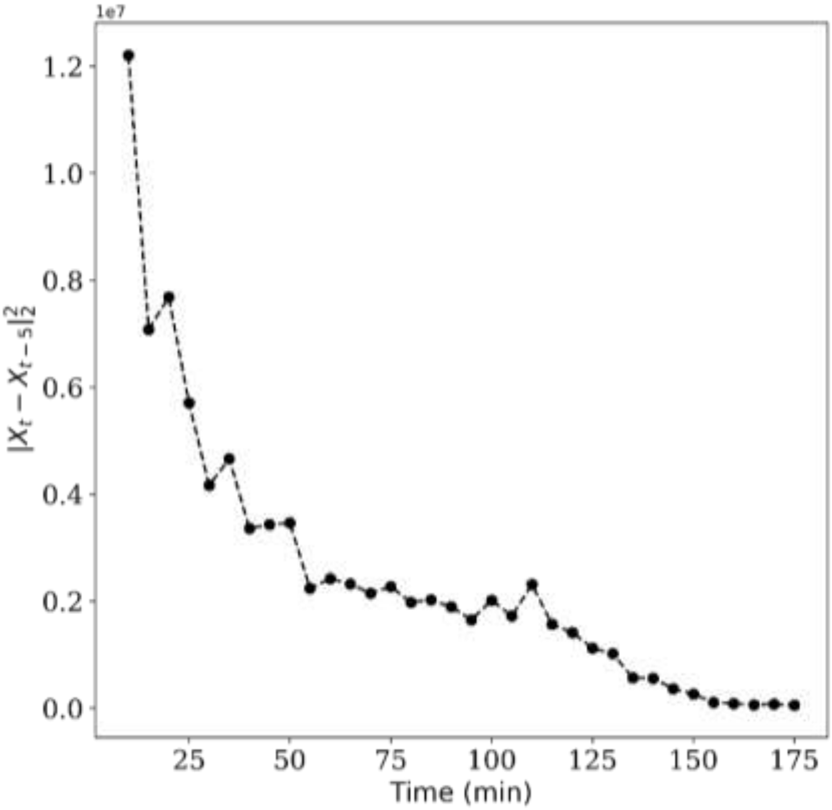

Figure S9. iPRISM convergence. As the number of measurements increases, the change in the matrix of pairwise DTW distances X, decreases monotonically. After 100 minutes, the change is negligible.

Table S1. Summary of demographic characteristics and pH values of collected clinical urine specimens.

	**Number of Samples**
**Age(years)**	
**0-39**	17(28%)
**40-79**	29(49%)
**>80**	14(23%)
**Gender**	
**male**	14(23%)
**female**	46(77%)
**pH**	
**5-5.5**	15(50%)
**6-6.5**	13(43%)
**>6.5**	2(7%)

Movie S1. Movie of *E. coli* cells growth on the silicon surface for the first 25 minutes.
